# Phenotyping Exertional Breathlessness Using Cardiopulmonary Cycle Exercise Testing in People With Chronic Airflow Limitation

**DOI:** 10.1016/j.chest.2025.02.033

**Published:** 2025-03-11

**Authors:** Magnus Ekström, Pei Zhi Li, Hayley Lewthwaite, Jean Bourbeau, Wan C. Tan, Dennis Jensen, Canadian Cohort Obstructive Lung Disease Collaborative Research Group

**Affiliations:** a Department of Clinical Sciences Lund, Respiratory Medicine, Allergology and Palliative Medicine, Faculty of Medicine, Lund University, Lund, Sweden; b Montreal Chest Institute, Montréal, QC, Canada; c Translational Research in Respiratory Diseases Program and Respiratory Epidemiology and Clinical Research Unit, McGill University Health Centre Research Institute, Montréal, QC, Canada; d Clinical Exercise and Respiratory Physiology Laboratory, Department of Kinesiology and Physical Education, Faculty of Education, McGill University, Montréal, QC, Canada; e Centre for Heart Lung Innovation, Department of Medicine, University of British Columbia, Vancouver, BC, Canada; f Centre of Research Excellence in Treatable Traits, College of Health, Medicine and Wellbeing, University of Newcastle, NSW, Australia; g Asthma and Breathing Research Program, Hunter Medical Research Institute, New Lambton, NSW, Australia

**Keywords:** dyspnea, exercise capacity, mechanisms, outcomes, ventilation

## Abstract

**Background:**

Exertional breathlessness is a cardinal symptom of people with chronic airflow limitation (CAL) and can be evaluated using cardiopulmonary exercise testing (CPET).

**Research Question:**

Does abnormally high exertional breathlessness in relationship to the rate of oxygen uptake (V′O_2_) and minute ventilation (V′_E_) indicate different underlying pathophysiologic mechanisms and clinical characteristics in people with CAL?

**Study Design and Methods:**

Analysis of people aged ≥ 40 years with CAL (FEV_1_ to FVC ratio after bronchodilation less than lower limit of normal) undergoing symptom-limited incremental cycle CPET in the Canadian Cohort Obstructive Lung Disease study. Using published normative references, breathlessness phenotypes at peak exercise were categorized as abnormal (Borg 0-10 scale intensity rating more than upper limit of normal) by V′O_2_ alone, abnormal by both V′O_2_ and V′_E_, or normal by both V′O_2_ and V′_E_. Exercise physiologic responses and clinical characteristics were compared between groups.

**Results:**

We included 325 people (44% female) with CAL (mean [SD]) FEV_1_, 75.4 [17.5] % predicted). Compared with the normal by both V′O_2_ and V′_E_ group (n = 237 [73%]), the abnormal by V′O_2_ only group (n = 29 [9%]) showed lower pulmonary diffusing capacity and greater exercise ventilatory inefficiency, whereas the abnormal by both V′O_2_ and V′_E_ group (n = 50 [15%]) showed even worse lung function, dynamic critical inspiratory constraints, and exertional breathlessness along with greater symptom burden in daily life, lower physical activity, and worse health status.

**Interpretation:**

Our results show that exertional breathlessness phenotyped in relationship to V′O_2_ and V′_E_ using normative reference equations enables multivariable analyses of underlying symptom mechanisms and associated clinical characteristics.

**Take-Home Points**

**Study Question:** Does abnormally high exertional breathlessness in relationship to oxygen uptake (V′O_2_) only or also to minute ventilation (V′_E_) indicate different underlying pathophysiologic mechanisms and clinical characteristics in people with chronic airflow limitation (CAL)?

**Results:** In 325 people (44% female) with mild to moderate CAL undergoing incremental cycle cardiopulmonary exercise testing, compared with those with normal breathlessness, the group with abnormal findings by V′O_2_ showed lower lung diffusing capacity and greater exercise ventilatory inefficiency, whereas the group with abnormal findings by V′O_2_ and V′_E_ showed even worse lung function, dynamic critical inspiratory constraints, and exertional breathlessness along with worse symptom burden, physical activity, and health status in daily life.

**Interpretation:** Our results show that phenotyping exertional breathlessness in relationship to level of exercise (V′O_2_) and ventilation (V′_E_) using normative reference equations informs on underlying symptom mechanisms and related clinical characteristics.

Breathlessness on exertion^1^^,^^2^ is a cardinal symptom in people with chronic airflow limitation (CAL) and is a leading cause of chronic illness and disability.^3^ The symptom trajectory often is progressive, leading to a vicious cycle of impaired physical activity, deconditioning, and worsening of breathlessness at progressively lower levels of exertion.^4^ Because many people often reduce their physical activity to avoid the symptom,^4^ the true severity of exertional breathlessness often is hidden and subsequently becomes underreported.^5^ Therefore, exertional breathlessness should be measured in relationship to a symptom stimulus such as at a standardized rate of oxygen uptake (V′O_2_) or minute ventilation (V′_E_).^6^

Cardiopulmonary exercise testing (CPET) is the gold standard method to evaluate exertional breathlessness and to identify the underlying mechanisms in clinical care and research.^7^, ^8^, ^9^ Normative reference equations are available^10^ and are validated in people with CAL^11^ to identify the presence of abnormally high exertional breathlessness (defined as a modified Borg 0-10 category ratio [Borg CR10] scale intensity rating of more than the predicted upper limit of normal) during symptom-limited incremental cycle CPET, in relationship to the level of exertion (power output or V′O_2_) or V′_E_.^10^^,^^12^ Even after accounting for differences in exercise capacity, abnormally high exertional breathlessness is associated strongly with increased mortality.^13^

Key pathophysiologic factors contributing to exertional breathlessness in people with CAL are: (1) abnormally high ventilatory drive resulting from exercise ventilatory inefficiency, gas exchange abnormalities, or both; (2) dynamic critical inspiratory constraints (CICs) resulting from dynamic lung hyperinflation and erosion of inspiratory reserve volume (IRV), which forces tidal volume (V_T_) to expand on the less compliant (nonlinear) portion of the respiratory system’s sigmoid pressure-volume curve (ie, close to total lung capacity [TLC]), where increased elastic loading and functional weakening of the inspiratory muscles occurs with attendant further increases in ventilatory drive; or both.^9^^,^^14^, ^15^, ^16^

To gain mechanistic insight into the pathophysiologic factors contributing to exertional breathlessness in CAL, it has been proposed that when breathlessness intensity ratings are (1) abnormally high in relationship to V′O_2_, but normal relative to V′_E_ during CPET, the main contributing mechanism likely is an abnormally high ventilatory demand, exercise ventilatory inefficiency, or both, or (2) abnormally high in relationship to both V′O_2_ and V′_E_ during CPET, a mechanistic role for CIC exists, either alone or in combination with abnormally high ventilatory demand or exercise ventilatory inefficiency.^9^ This proposed interpretation strategy is supported by the results of multiple small mechanistic studies in selected groups of symptomatic outpatients with chronic pulmonary disorders,^16^ but has not been assessed in a large group of people with mostly asymptomatic mild CAL randomly sampled from the general population.

Recently developed normative reference equations for exertional breathlessness during CPET provide a novel opportunity to study the phenotype of abnormally high exertional breathlessness (in relationship to V′O_2_, V′_E_, or both) compared with people with exertional breathlessness within the normal predicted range(s) in the population, in relationship to physiologic and clinical characteristics. The aim of this study was to investigate how the phenotype of exertional breathlessness intensity response, categorized as abnormally high by V′O_2_ only, abnormally high by both V′O_2_ and V′_E_, or normal by both V′O_2_ and V′_E_, relates to underlying pathophysiologic mechanisms and clinical characteristics in a population sample of people with CAL.

## Study Design and Methods

### Study Design and Population

This was an analysis of the prospective, population-based Canadian Cohort Obstructive Lung Disease (CanCOLD) study.^17^ Participants were noninstitutionalized male or female adults aged 40 years or older originally identified with random telephone digit dialing across 9 communities in Canada.^17^ All participants provided written informed consent before completing study assessments. The research ethics board for each participating institution approved the study protocol. The study was approved by the respective university and institutional ethical review boards: McGill University Health Centre Research Ethics Board, 09-025-BMB-t (Montreal); University of British Columbia/Providence Health Care Research Ethics Board, P05-006 (Vancouver); University Health Network Research Ethics Board, 06-0421-B (Toronto); Capital Health Research Ethics Board, CDHA-RS/2007-255 (Halifax); Conjoint Health Research Ethics Board, ID21258 (Calgary); Department of Medicine-1240-09 (Kingston); 2009519-01H (Ottawa); Bio-Research Ethics Board 09-162 (Saskatoon); Comité d'éthique de la recherche 20459 (Quebec City). This CanCOLD substudy is reported in accordance with the Strengthening the Reporting of Observational Studies in Epidemiology statement.^18^

**Figure 1:**
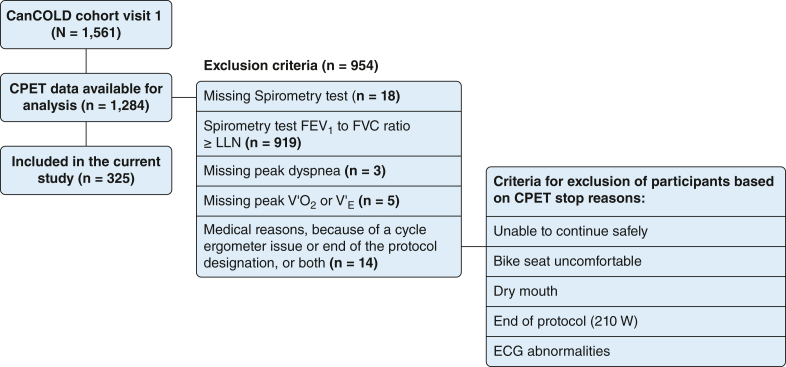
Participant flow chart. CanCOLD = Canadian Cohort Obstructive Lung Disease; CPET = cardiopulmonary exercise testing; LLN = lower limit of normal; V′_E_ = minute ventilation; V′O_2_ = rate of oxygen uptake; W = power output (watts).

Eligibility criteria for this analysis were (1) CAL defined as FEV_1_ to FVC ratio after bronchodilation of less than lower limit of normal and (2) performed CPET during the CanCOLD baseline visit without adverse events and with data including peak breathlessness intensity (Borg CR10 scale) rating (Fig 1). The present data were used previously for validating the normative reference equations of exertional breathlessness during CPET,^11^ but to our knowledge, the present analyses by exertional breathlessness phenotype are novel and have not been reported previously.

### Assessments and Procedures

Participants self-reported sociodemographics and health information (eg, smoking history and presence of physician-diagnosed health conditions) via structured interview with a trained researcher, whereas health status was assessed using the St George’s Respiratory Questionnaire^19^ and COPD Assessment Test.^20^ Impact of breathlessness on daily life activity was assessed using the ordinal Medical Research Council breathlessness scale ranging from 1 to 5.^21^ Physical activity was self-reported using the Community Healthy Activities Model Program for Seniors questionnaire.^22^ Spirometry after bronchodilation (200 μg albuterol), diffusing capacity of the lungs for carbon monoxide, and plethysmographic lung volumes were assessed using automated equipment in accordance with published standards.^17^^,^^23^^,^^24^ Predicted lung function values were calculated using Global Lung Function Initiative references.^25^, ^26^, ^27^

#### Cardiopulmonary Exercise Testing

CPET was performed in accordance with recognized guidelines on an electronically braked cycle ergometer using a computerized CPET system.^28^ The CPET protocol was standardized across sites and included a steady state baseline period before exercise of 3 to 10 minutes, followed by 1 minute of unloaded pedalling, and then a 10-W/min increase in power output (starting at 10 W) until symptom limitation.^29^

Gas exchange and breathing pattern parameters were collected breath-by-breath with participants breathing through a mouthpiece and flow transducer while wearing a nose clip. Heart rate and rhythm were assessed continuously by 12-lead ECG, and peripheral oxyhemoglobin saturation was monitored by finger pulse oximetry. At rest, every 2 minutes during exercise, and at peak exercise, BP was assessed, and participants performed maximal voluntary inspiratory capacity (IC) maneuvers^30^ and rated the intensity of their perceived breathlessness and leg discomfort using the Borg CR10 scale.^31^ Before CPET, breathlessness was defined for each participant as breathing discomfort, and leg discomfort was defined as the level of discomfort experienced during pedalling; and participants were familiarized with the Borg CR10 scale such that 0 represented no breathing (or leg) discomfort and 10 represented the most severe breathing (leg) discomfort that you have ever experienced or can imagine experiencing. Peak power output (in watts) was taken as the highest power output a participant was able to sustain for ≥ 30 seconds, whereas peak V′O_2_ and peak V′_E_ were taken as the average of the last 30 seconds of loaded pedalling.

#### Physiologic Responses to CPET

Physiologic variables evaluated during CPET included exercise capacity as peak watts and peak V′O_2_; dynamic hyperinflation as change in IC from baseline before exercise to peak exercise indexed to peak V′_E_ (ΔIC/V′_E_; with lower values indicating greater dynamic hyperinflation); ventilatory inefficiency as the nadir of the ventilatory equivalent for CO_2_ (nadir ventilatory equivalent for CO_2_ [V′_E_/V′CO_2_]), identified as the lowest 30-second average data point observed during CPET; CIC as the (1) V_T_ to IC ratio and (2) end-inspiratory lung volume (EILV) to TLC ratio, with both ratios indexed to peak V′_E_ (V_T_ to IC ratio per V′_E_ and EILV to TLC ratio per V′_E_), as well as (3) the IRV at peak exercise.

Unless indicated otherwise, all physiologic variables and Borg CR10 scale symptom ratings used in the analyses were values obtained at the symptom-limited peak of exercise. Predicted values for peak watts, peak V′O_2_, peak V′_E_, and nadir V′_E_/V′CO_2_ were calculated using CanCOLD references.^29^

#### Abnormal Exertional Breathlessness Phenotypes

Abnormally high exertional breathlessness was defined as a Borg CR10 scale breathlessness intensity rating at peak exercise of more than upper limit of normal in relationship to peak V′O_2_ and peak V′_E_, using published CanCOLD normative reference equations.^10^

### Statistical Analyses

Characteristics were summarized using mean (SD) and median with range or interquartile range for continuous variables, as appropriate. Categorical variables were expressed as frequencies and percentages. Participants with exertional breathlessness categorized as abnormally high by V′_E_ only were not included in analyses because few people showed this phenotype (n = 9 [2.8%]). Physiologic and clinical variables were compared among the 3 breathlessness phenotype groups using the analysis of variance for continuous variables and χ^2^ tests for categorical variables, with Bonferroni adjustment for the post hoc multiple comparisons. Associations between the breathlessness phenotype groups and physiologic and clinical characteristics were analyzed using linear regression for continuous and logistic regression for dichotomous variables, unadjusted and adjusted for age, sex, BMI, and pack-years of smoking. Statistical significance was defined as a 2-sided *P* value of < .05. Statistical analyses were conducted using the SAS version 9.4 software (TS1M5; SAS Institute, Inc.).

## Results

A total of 325 people (44% female) with CAL were included (Fig 1). Participants in this population sample had mostly mild to moderate airflow limitation (mean [SD] FEV_1_, 75.4 [17.5] % predicted) and 50% did not take any respiratory medication (Table 1).

**Table 1:** Characteristics of Participants (N = 325)

Characteristic	All Participants
Age, y	
Mean (SD)	64.3 (10.2)
Interquartile range	40.0-89.0
Female sex	143 (44.0%)
BMI, kg/m^2^	27.2 (4.9)
Smoking status	
Never	92 (28.3%)
Former	163 (50.2%)
Current	70 (21.5%)
Pack-years of smoking	25.8 (24.8)
Hypertension	102 (31.4%)
Physician-diagnosed COPD	124 (38.2%)
Physician-diagnosed asthma	138 (42.5%)
Any respiratory medication	162 (49.8%)
Self-reported outcomes	
MRC breathlessness scale rating	
1	148 (45.5%)
2	133 (40.9%)
≥ 3	33 (10.2%)
CAT score	
Total	8.9 (6.9)
Total ≥ 10	128 (39.5%)
SGRQ total score	18.6 (16.1)
CHAMPS, kcal/wk	
Moderate and greater intensity	2,348 (2,464)
All activities	4,128 (2,988)
Lung function at rest	
FEV_1_, % predicted	75.4 (17.5)
FVC, % predicted	101.9 (19.2)
FEV_1_ to FVC ratio, %	57.3 (8.1)
TLC, % predicted	109.9 (15.1)
IC, % predicted	95.1 (22.6)
RV, % predicted	144.3 (42.1)
RV per TLC, %	0.44 (0.10)
FRC, % predicted	121.9 (28.6)
FRC per TLC, %	0.58 (0.09)
Dlco	
% Predicted	87.6 (23.2)
< LLN	96 (30.7%)
CPET parameters at peak exercise	
Power output, W	
% Predicted	81.2 (24.9)
< LLN	92 (28.3%)
V′O_2_	
% Predicted	82.9 (23.1)
< LLN	71 (21.8%)
V′_E_	
% Predicted	83.5 (24.7)
Per MVV (FEV_1_ × 35)	76.1 (20.4)
V′_E_/V′CO_2_	
Nadir	32.0 (6.8)
Nadir > 34	99 (30.5)
IC, L	
Mean (SD)	2.8 (0.8)
Change	–0.2 (0.4)
IRV, L	
Mean (SD)	0.8 (0.4)
≤ 0.75	166 (53.5%)
EILV, L	5.9 (1.3)
V_T_ to IC ratio, %	71.2 (12.3)
EILV to TLC ratio, %	88.7 (5.8)
V_T_ to IC ratio per V′_E_	1.41 (0.53)
EILV to TLC ratio per V′_E_	1.81 (0.73)
ΔIC/V′_E_	–0.004 (0.009)
Breathlessness, Borg CR10 scale	5.0 (4.0-7.0)
Leg discomfort, Borg CR10 scale	7.0 (4.0-8.0)
Breathlessness per V′O_2_, Borg CR10 scale/L/min	3.75 (2.30)
Breathlessness per V′_E_, Borg CR10 scale/L/min	0.10 (0.06)
Leg discomfort per V′O_2_, Borg CR10 scale/L/min	4.44 (2.54)
Reasons for stopping CPET	
Breathlessness	60 (18.9%)
Leg discomfort	129 (40.7%)
Breathlessness and leg discomfort	68 (21.5%)
Other	60 (18.9%)

Data are presented as No. (%), mean (SD), or median (interquartile ranges) unless otherwise specified. Borg CR10 = Borg 0-10 category ratio; CAT = COPD Assessment Test; CHAMPS = Physical Activity Questionnaire for Older Adults; CPET = cardiopulmonary exercise testing; ΔIC/V′_E_ = change in inspiratory capacity from baseline before exercise to peak exercise indexed to peak minute ventilation; Dlco = diffusion capacity of the lungs for carbon monoxide; EILV = end-inspiratory lung volume; FRC = functional residual capacity; IC = inspiratory capacity; IRV = inspiratory reserve volume; LLN = lower limit of normal; MRC = Medical Research Council; MVV = maximum voluntary ventilation (estimated); RV = residual volume; SGRQ = St. George’s Respiratory Questionnaire; TLC = total lung capacity; V′CO_2_, rate of CO_2_ production; V′_E_ = minute ventilation; V′_E_/V′CO_2_ = ventilatory equivalent for CO_2_; V′O_2_ = rate of oxygen uptake; V_T_ = tidal volume.

The prevalence of abnormally high exertional breathlessness overall (in relationship to V′O_2_ or to V′_E_) was 27.1%, which was comparable with the prevalence of abnormally low peak power output (28.3%) and peak V′O_2_ (21.8%) (Table 1). The prevalence of abnormal findings by V′O_2_ only was 9% (n = 29), and the prevalence of abnormal findings by both V′O_2_ and V′_E_ was 15.4% (n = 50).

Compared with the group with normal findings by both V′O_2_ and V′_E_ (72.9%), people with abnormally high exertional breathlessness (abnormal by V′O_2_ only and abnormal by both V′O_2_ and V′_E_ groups) showed more severely impaired peak exercise capacity as measured by peak V′O_2_ and lower diffusing capacity of the lungs for carbon monoxide (% predicted and proportion less than the lower limit of normal); however, only the group with abnormal findings by V′O_2_ only showed significantly higher V′_E_/V′CO_2_ (ie, greater ventilatory inefficiency) compared with the group with normal findings by both V′O_2_ and V′_E_. The group with abnormal findings by both V′O_2_ and V′_E_ showed significantly greater abnormalities in spirometry (lower FEV_1_ and FVC % predicted and lower FEV_1_ to FVC ratio) and plethysmographic lung volumes (IC % predicted, residual volume in % of TLC) compared with the group with normal findings by both V′O_2_ and V′_E_ (Table 2).

**Table 2:** Clinical and Physiologic Factors by Exertional Breathlessness Phenotype

Factor	Normal Findings by Both V′O_2_ and V′_E_	Abnormal Findings by V′O_2_ Alone	Abnormal Findings by V′O_2_ and V′_E_	*P* Value (Among the 3 Groups
No. of patients (%)	237 (72.9%)	29 (8.9%)	50 (15.4%)	
Age, y				< .001
Mean (SD)	62.9 (9.7)^a^	65.3 (10.8)^a^	69.6 (9.7)^a^	
Interquartile range	40.0-87.0^a^	41.0-82.0^a^	40.0-88.0^a^	
Female sex	100 (42.2%)	13 (44.8%)	29 (58.0%)	.124
BMI, kg/m^2^	27.2 (4.6)	25.3 (5.9)	27.4 (5.3)	.021
Smoking status				
Never	70 (29.5%)	8 (27.6%)	9 (18.0%)	.252
Former	117 (49.4%)	14 (48.3%)	28 (56.0%)	.677
Current	50 (21.1%)	7 (24.1%)	13 (26.0%)	.723
Pack-years of smoking	24.4 (23.6)	30.8 (24.0)	31.4 (29.6)	.154
Hypertension	64 (27.0%)	11 (37.9%)	21 (42.0%)	.072
Physician-diagnosed COPD	80 (33.8%)^a^	11 (37.9%)	28 (56.0%)^a^	.013
Physician-diagnosed asthma	99 (41.8%)	8 (27.6%)	26 (52.0%)	.104
Any respiratory medication	116 (48.9%)	10 (34.5%)	31 (62.0%)	.056
Self-reported outcomes				
MRC dyspnea scale breathlessness rating				
1, n (%)	119 (50.2%)^a^	12 (41.4%)	10 (20.0%)^a^	< .001
2, n (%)	96 (40.5%)	13 (44.8%)	23 (46.0%)	.728
≥ 3, n (%)	17 (7.2%)^a^	1 (3.4%)	14 (28.0%)^a^	< .001
CAT score				
Total	8.0 (6.1)^a^	10.1 (7.1)	12.8 (8.7)^a^	< .001
Total ≥ 10	78 (33.1%)^a^	15 (51.7%)	31 (62.0%)^a^	< .001
SGRQ total score	16.0 (14.1)^a^	20.4 (15.4)	29.4 (20.8)^a^	< .001
CHAMPS, kcal/wk				
Moderate and greater intensity	2,513 (2,522)^a^	2,338 (2,336)	1,276 (1,724)^a^	< .001
All activities	4,302 (3,057)^a^	4,141.1 (2,470)	2,940.7 (2,369)^a^	.003
Lung function at rest				
FEV_1_, % predicted	77.9 (17.3)^a^	73.6 (9.2)	64.8 (18.9)^a^	< .001
FVC, % predicted	103.5 (18.8)^a^	100.7 (12.6)	94.5 (21.1)^a^	.009
FEV_1_ to FVC ratio, %	58.4 (7.4)^a^	56.8 (7.8)	52.7 (9.3)^a^	< .001
TLC, % predicted	110.2 (14.9)	108.8 (15.2)	109.3 (16.3)	.859
IC, % predicted	96.2 (23.3)	95.7 (17.8)	88.5 (22.5)	.029
RV, % predicted	144.0 (41.4)	140.4 (41.5)	148.6 (45.6)	.544
RV per TLC, %	0.43 (0.10)^a^	0.43 (0.09)	0.49 (0.11)^a^	< .001
FRC, % predicted	120.8 (27.4)	120.6 (34.6)	129.6 (29.0)	.211
FRC per TLC, %	0.57 (0.08)^a^	0.58 (0.11)	0.64 (0.08)^a^	< .001
Dlco				
% Predicted	90.6 (22.4)^a^^,^^b^	73.1 (19.6)^b^	79.1 (22.8)^a^	< .001
< LLN	57 (25.0%)^a^^,^^b^	17 (58.6%)^b^	22 (45.8%)^a^	< .001
CPET parameter at peak exercise				
Power output, W				
% Predicted	83.1 (25.6)	75.1 (19.1)	74.3 (24.0)	.041
< LLN	61 (25.7%)	12 (41.4%)	18 (36.0%)	.101
V′O_2_				
% Predicted	86.4 (23.1)^a^^,^^b^	70.6 (21.3)^b^	72.6 (19.0)	< .001
< LLN	42 (17.7%)^a^^,^^b^	11 (37.9%)^b^	17 (34.0%)	.004
V′_E_,				
% Predicted	86.3 (25.7)^a^	85.6 (22.3)	69.8 (16.9)^a^	< .001
Per MVV (FEV_1_ × 35)	76.0 (20.8)	79.0 (21.1)	75.6 (18.4)	.623
V′_E_/V′CO_2_				
Nadir	31.5 (6.6)^b^	36.0 (6.9)^b^	32.9 (6.9)	< .001
Nadir > 34	64 (27.0%)^b^	17 (58.6%)^b^	17 (34.0%)	.002
IC, L				
Mean (SD)	2.9 (0.8)^a^	2.8 (0.9)^b^	2.2 (0.7)^a^^,^^b^	< .001
Change	–0.2 (0.4)	–0.1 (0.4)	–0.3 (0.4)	.236
IRV, L				
Mean (SD)	0.8 (0.4)^a^	0.8 (0.4)^b^	0.6 (0.3)^a^^,^^b^	.015
≤ 0.75	117 (52.2%)^a^	16 (57.1%)	37 (75.5%)^a^	.011
EILV, L	6.0 (1.3)	5.9 (1.4)	5.5 (1.3)	.065
V_T_ to IC ratio, %	71.4 (12.2)	69.2 (12.1)	70.5 (12.5)	.634
EILV to TLC, %	88.5 (5.8)	87.5 (6.8)	90.0 (4.9)	.347
V_T_ to IC ratio per V′_E_	1.33 (0.47)^a^	1.25 (0.33)	1.86 (0.63)^a^	< .001
EILV to TLC ratio per V′_E_	1.69 (0.65)^a^	1.61 (0.55)	2.42 (0.84)^a^	< .001
ΔIC/V′_E_	–0.004 (0.007)^a^	–0.002 (0.008)	–0.008 (0.013)^a^	.010
Breathlessness	4.0 (3.0,6.0)^a^	6.0 (5.0,7.0)^a^	8.0 (7.0,9.0)^a^	< .001
Leg discomfort	5.0 (4.0-7.0)^a^^,^^b^	7.0 (6.0-9.0)^b^	7.0 (5.0-9.0)^a^	< .001
Breathlessness per V′O_2_	2.84 (1.22)^a^^,^^b^	5.45 (3.41)^b^	6.97 (2.15)^a^	< .001
Breathlessness per V′_E_	0.08 (0.03)^a^	0.11 (0.03)^a^	0.20 (0.06)^a^	< .001
Leg discomfort per V′O_2_	3.74 (1.98)^a^^,^^b^	6.39 (3.62)^a^	6.55 (2.61)^b^	< .001
Reasons for stopping CPET				
Breathlessness	26 (11.3%)^a^	6 (20.7%)	25 (51.0%)^a^	< .001
Leg discomfort	106 (46.1%)^a^	12 (41.4%)	8 (16.3%)^a^	< .001
Breathlessness and leg discomfort	46 (20.0%)	8 (27.6%)	14 (28.6%)	.306
Other	52 (22.6%)^a^	3 (10.3%)	2 (4.1%)^a^	.003

Data are presented as No. (%), mean (SD), or median (interquartile ranges) unless otherwise specified. CAT = COPD Assessment Test; CHAMPS = Community Healthy Activities Model Program for Seniors questionnaire; CPET = cardiopulmonary exercise testing (cycle); ΔIC/V′_E_ = change in IC from baseline before exercise to peak exercise indexed to peak V′_E_; Dlco = diffusion capacity of the lungs for carbon monoxide; EILV = end-inspiratory lung volume; FRC = functional residual capacity; IC = inspiratory capacity; IRV = inspiratory reserve volume; LLN = lower limit of normal; MRC = Medical Research Council; MVV = maximal voluntary ventilation; RV = residual volume; SGRQ = St George’s Respiratory Questionnaire; TLC = total lung capacity; V′CO_2_ = rate of exhaled CO_2_; V′_E_ = minute ventilation; V′_E_/V′CO_2_ = ventilatory equivalent for CO_2_; V′O_2_ = rate of oxygen uptake; V_T_ = tidal volume.

a Group means with same letter "a" are significantly different from each other after Bonferroni adjustment for multiple comparisons (*P* < .05).

b Group means with same letter "b" are significantly different from each other after Bonferroni adjustment for multiple comparisons (*P* < .05).

Compared with both the group with normal findings by both V′O_2_ and V′_E_ and the group with abnormal findings by V′O_2_ only, the group with abnormal findings by both V′O_2_ and V′_E_ reached a lower peak V′_E_ and had more severe CIC, as indicated by higher peak V_T_ to IC ratio per V′_E_, EILV to TLC ratio per V′_E_, ΔIC/V′_E_, and a higher proportion of people with a peak IRV of ≤ 0.75 L (Table 2). Compared with the group with normal findings by both V′O_2_ and V′_E_, the group with abnormal findings by V′O_2_ and V′_E_ also were more likely to stop exercise because of intolerable breathlessness (and leg discomfort); to self-report greater respiratory symptom burden, poorer health status, and lower daily physical activity; and to have physician-diagnosed COPD (Table 2). The findings were similar in regression analyses when adjusted for between-group differences in age, sex, BMI, and cigarette pack-years (e-Table 1).

## Discussion

### Main Findings and Interpretations

This study showed that, in a population sample of people with CAL, phenotyping exertional breathlessness as abnormally high in relationship to peak V′O_2_ alone or together with peak V′_E_ using incremental cycle CPET provides mechanistic insight into the pathophysiologic factors responsible for the exaggerated exertional symptom response, with implications for multiple clinical and patient-reported responses and characteristics.

Recently developed reference equations enable, for the first time, exertional breathlessness responses to be evaluated for (ab)normality in relationship to key physiologic metrics (power output, V′O_2_, V′_E_, or a combination thereof)^10^ for characterization and comparisons between individuals or populations. Using this approach, we identified that, although those with abnormally high exertional breathlessness for peak V′O_2_ alone (those with abnormal findings by V′O_2_) or together with peak V′_E_ (those with abnormal findings by V′O_2_ and V′_E_) have similarly impaired peak exercise capacity (peak V′O_2_) and diffusing capacity of the lungs for carbon monoxide, the abnormal findings by V′O_2_ group demonstrated a mechanistic pattern consistent with greater exercise ventilatory inefficiency (higher V′_E_/V′CO_2_ nadir), whereas the group with abnormal findings by V′O_2_ and V′_E_ demonstrated a mechanistic pattern consistent with more severe CICs (higher peak V_T_ to IC ratio per V′_E_, EILV to TLC ratio per V′_E_, ΔIC/V′_E_, and a higher proportion of people with a peak IRV ≤ 0.75 L). Importantly, this latter CIC phenotype was associated with a greater symptom burden, worse health status, and lower physical activity levels. Because CIC is a treatable trait—and interestingly, about one-half of people with CAL in the present population were not treated with any bronchodilator—the present findings highlight a population whose breathlessness could be detected using standardized exercise testing and who could benefit from optimized treatment. Clinically, the present findings validate the CPET interpretation strategy recommended by Stickland et al^9^ to gain mechanistic insight into the pathophysiologic factors most responsible for an individual’s exertional breathlessness.

Consistent with the results of Guenette et al,^32^ our findings suggest that the abnormally high breathlessness response to exercise in people with CAL is not driven by dynamic hyperinflation per se, but rather likely reflects the awareness of an abnormally high ventilatory drive resulting from exercise ventilatory inefficiency, severe CICs, or both. This is supported by the observation that exertional breathlessness was much more severe in each of the groups with abnormal findings by V′O_2_ only and by V′O_2_ and V′_E_ (compared with the group with normal findings by both V′O_2_ and V′_E_), although ΔIC/V′_E_ was not different in the group with abnormal findings by V′O_2_ vs the group with normal findings by both V′O_2_ and V′_E_.

### Strengths and Limitations

A strength of the present study is the inclusion of a relatively large group of people with CAL from the well-characterized CanCOLD cohort, which provides unique population data on self-reported outcomes using validated instruments combined with detailed physiologic assessments including cycle CPET, the golden standard method to assess exertional breathlessness.^7^, ^8^, ^9^ Exertional breathlessness intensity responses were evaluated in relationship to peak V′O_2_ and peak V′_E_ using normative reference equations developed with CPET data from CanCOLD^10^ and have been validated internally and externally in people with CAL.^11^ Compared with previous smaller exercise physiologic studies of participants recruited in clinical care, the use of a population sample independent of clinical contact decreases the risk of selection bias.

A limitation is the inclusion of relatively few people with severe CAL, which reflects the severity distribution of airflow limitation in the general population.^33^ The present findings pertain mostly to people with mild to moderate airflow limitation. Arterial blood gases were not assessed, which would be helpful to identify the underlying mechanism(s) of the elevated V′_E_/V′CO_2_. However, increased dead space was reported as the most consistent gas exchange abnormality contributing to an exaggerated ventilatory response to exercise in mild COPD.^34^ The prevalence of abnormally high exertional breathlessness (in relationship to peak V′O_2_, peak V′_E_, or both) in this population sample was approximately 27% and was similar to that observed for abnormally low peak exercise capacity (peak power output and V′O_2_ less than the lower limit of normal of 28% and 22%, respectively), which helps to validate our findings.

## Interpretation

The present results have important implications. For health care providers and clinical physiology laboratories, the present findings show how the exertional breathlessness intensity response can be phenotyped using symptom-limited incremental cycle CPET and published normative reference equations^10^ to detect the presence and level of abnormal exertional breathlessness and to inform on the underlying pathophysiologic mechanisms. Because many people are referred to CPET for evaluation of exertional breathlessness of unknown cause, quantifying and characterizing the breathlessness response to CPET could aid clinical interpretation and differential diagnostics with implications for more targeted (individualized) therapeutic intervention(s). Using the reference equations can improve detection and grading of exertional breathlessness in people with COPD.^35^ Indeed, a recent analysis from CanCOLD showed that the widely used modified Medical Research Council and COPD Assessment Test questionnaires fail to detect the presence of abnormally high exertional breathlessness (assessed using CPET normative equations) in people with mostly mild to moderate COPD, with a sensitivity of only 12%.^35^ Use of standardized exercise testing with normative reference equations, through improved symptom grading, may optimize COPD management including inhalation therapy through reducing overtreatment and undertreatment and may be useful to differentiate respiratory from other potentially treatable causes of exertional breathlessness (eg, deconditioning). A next research step is to investigate how the identification of exertional breathlessness abnormality phenotypes can be used to improve clinical evaluation and management of people with activity-related breathlessness of unknown cause and in people with different cardiorespiratory diseases.

## Funding/Support

The CanCOLD study (ClinicalTrials.gov Identifier: NCT00920348) has received support from the 10.13039/501100021743Canadian Respiratory Research Network, the Canadian Institutes of Health Research [CIHR/Rx&D Collaborative Research Program Operating Grant 93326], the Respiratory Health Research Network of the Fonds de la Recherche en Santé du Québec, the Foundation of the 10.13039/100014131McGill University Health Centre, and industry partners, including: 10.13039/100008207AstraZeneca Canada, Ltd., Boehringer Ingelheim Canada, Ltd., GlaxoSmithKline (GSK) Canada, Ltd., Novartis, Almirall, Merck, Nycomed, Pfizer Canada, Ltd., and Theratechnologies. M. E. was supported by an unrestricted grant from the Swedish Research Council [Grant: 2019-02081]. D. J. holds a Canada Research Chair, Tier II, in Clinical Exercise & Respiratory Physiology from the 10.13039/501100000024Canadian Institutes of Health Research.

## Financial/Nonfinancial Disclosures

The authors have reported to *CHEST* the following: J. B. and W. C. T. report receiving institutional funding for the CanCOLD study from Astra Zeneca Canada, Ltd., Boehringer-Ingelheim Canada, Ltd., GlaxoSmithKline Canada, Ltd., Merck, Novartis Pharma Canada, Inc., as well as Nycomed Canada, Inc. (W. C. T.), Pfizer Canada, Ltd. (W. C. T.), Trudell (J. B.), and Grifolds (J. B.). None declared (M. E., P. Z. L., H. L., D. J.).

## Supplementary Data

e-Online Data
