# Supplementary material for: Phenotyping Exertional Breathlessness Using Cardiopulmonary Cycle Exercise Testing in People With Chronic Airflow Limitation
Source: Chest. 2025 Mar 11;168(2):379–89. doi: 10.1016/j.chest.2025.02.033 (PMC12405935; doi:10.1016/j.chest.2025.02.033)
Supplement: e-Online Data [file mmc1.docx]

**SUPPLEMENTAL MATERIAL**

**Phenotyping exertional breathlessness and underlying pathophysiological mechanisms using cardiopulmonary cycle exercise testing in people with chronic airflow limitation**

**e-Table 1.** Associations with physiologic and self-reported responses for each exertional breathlessness phenotype

|  | **Univariable analysis** | | | **Multivariable analysis** | | |
| --- | --- | --- | --- | --- | --- | --- |
| **Factor** | **B-normal**  (Exertional breathlessness normal by peak V’O_2_ and peak V’_E_) | **B-V’O_2_**  (Exertional breathlessness abnormal by peak V’O_2_ alone) | **B-V’O_2_+V’_E_**  (Exertional breathlessness abnormal by both peak V’O_2_ and peak V’_E_) | **B-normal**  (Exertional breathlessness normal by peak V’O_2_ and peak V’_E_) | **B-V’O_2_**  (Exertional breathlessness abnormal by peak V’O_2_ alone) | **B-V’O_2_+V’_E_**  (Exertional breathlessness abnormal by both peak V’O_2_ and peak V’_E_) |
| **Lung function at rest** |  | **Crude β/OR (95% CI)** | **Crude β/OR (95% CI)** |  | **Adj. β/OR (95% CI)** | **Adj. β/OR (95% CI)** |
| FEV_1_, %pred | Ref | -4.62 (-11.09, 1.85) | -13.11 (-18.23, -8.00) | Ref | -3.67 (-10.05, 2.70) | -11.44 (-16.58, -6.30) |
| FVC, %pred | Ref | -2.91 (-9.98, 4.16) | -9.19 (-14.78, -3.59) | Ref | -2.34 (-9.37, 4.70) | -7.67 (-13.34, -1.99) |
| FEV_1_/FVC, % | Ref | -1.54 (-4.54, 1.45) | -5.75 (-8.13, -3.37) | Ref | -0.38 (-3.17, 2.40) | -3.73 (-6.06, -1.41) |
| TLC, %pred | Ref | -1.78 (-7.51, 3.96) | -2.13 (-6.77, 2.51) | Ref | -1.81 (-7.54, 3.92) | -1.35 (-6.11, 3.41) |
| IC, %pred | Ref | -1.26 (-10.01, 7.50) | -7.31 (-14.50, -0.13) | Ref | -0.90 (-9.65, 7.85) | -6.16 (-13.52, 1.19) |
| RV/TLC, % | Ref | 0.01 (-0.03, 0.04) | 0.06 (0.03, 0.09) | Ref | -0.00 (-0.04, 0.03) | 0.03 (-0.00, 0.05) |
| FRC/TLC, % | Ref | 0.01 (-0.02, 0.04) | 0.06 (0.04, 0.09) | Ref | -0.00 (-0.03, 0.03) | 0.04 (0.02, 0.06) |
| D_L_CO, %pred | Ref | -17.2 (-25.31, -9.08) | -12.94 (-19.48, -6.40) | Ref | -15.25 (-22.91, -7.59) | -10.32 (-16.64, -4.01) |
| D_L_CO < LLN | Ref | 4.43 (1.93, 10.17) | 2.81 (1.43, 5.51) | Ref | 4.31 (1.82, 10.21) | 2.61 (1.29, 5.29) |
| **CPET parameter at peak exercise** |  |  |  |  |  |  |
| Power output, %pred | Ref | -8.52 (-18.12, 1.08) | -9.73 (-17.31, -2.14) | Ref | -6.20 (-15.35, 2.95) | -5.74 (-13.11, 1.62) |
| W < LLN | Ref | 2.01 (0.90, 4.48) | 1.66 (0.87, 3.19) | Ref | 1.83 (0.81, 4.16) | 1.25 (0.62, 2.52) |
| V’O_2_, %pred | Ref | -17.46 (-25.94, -8.97) | -14.26 (-20.97, -7.55) | Ref | -15.66 (-23.87, -7.44) | -11.62 (-18.24, -5.00) |
| V’O_2_ < LLN | Ref | 3.11 (1.33, 7.28) | 2.57 (1.29, 5.13) | Ref | 2.93 (1.25, 6.87) | 2.14 (1.04, 4.40) |
| V’_E_, %pred | Ref | -2.71 (-11.98, 6.55) | -16.48 (-23.80, -9.17) | Ref | -2.08 (-11.39, 7.24) | -15.57 (-23.08, -8.07) |
| V'_E_%MVV (FEV_1_ x 35) | Ref | 1.42 (-6.39, 9.23) | -0.19 (-6.37, 5.99) | Ref | 1.35 (-6.51, 9.21) | -1.31 (-7.89, 5.27) |
| Nadir V’_E_/V’CO_2_ | Ref | 4.53 (2.18, 6.88) | 2.23 (0.36, 4.10) | Ref | 3.03 (1.03, 5.02) | -0.72 (-2.39, 0.96) |
| Nadir V'_E_/V'CO_2_ > 34 | Ref | 4.73 (2.04, 10.99) | 1.65 (0.83, 3.28) | Ref | 3.34 (1.29, 8.64) | 0.73 (0.32, 1.67) |
| Nadir V'_E_/V'CO_2_ > ULN | Ref | 5.51 (2.29, 13.28) | 1.74 (0.81, 3.74) | Ref | 5.01 (2.06, 12.20) | 1.28 (0.56, 2.95) |
| IC | Ref | -0.22 (-0.52, 0.07) | -0.64 (-0.88, -0.41) | Ref | -0.08 (-0.31, 0.15) | -0.31 (-0.50, -0.12) |
| ∆ IC | Ref | 0.07 (-0.08, 0.23) | -0.08 (-0.20, 0.04) | Ref | 0.05 (-0.10, 0.20) | -0.03 (-0.15, 0.10) |
| IRV | Ref | 0.02 (-0.14, 0.18) | -0.20 (-0.32, -0.08) | Ref | 0.05 (-0.11, 0.20) | -0.13 (-0.26, -0.01) |
| EILV | Ref | -0.17 (-0.66, 0.32) | -0.51 (-0.89, -0.14) | Ref | -0.12 (-0.50, 0.27) | -0.15 (-0.46, 0.17) |
| VT%IC/V'_E_ | Ref | -0.03 (-0.22, 0.16) | 0.53 (0.39, 0.68) | Ref | -0.10 (-0.26, 0.06) | 0.35 (0.22, 0.49) |
| EILV%TLC/V'_E_ | Ref | -0.01 (-0.28, 0.25) | 0.72 (0.52, 0.93) | Ref | -0.11 (-0.33, 0.12) | 0.44 (0.26, 0.62) |
| ∆IC/V'_E_ | Ref | 0.002 (-0.002, 0.005) | -0.004 (-0.007, -0.002) | Ref | 0.001 (-0.002, 0.005) | -0.003 (-0.006, -0.000) |
| % with V_T_%IC ≥ 70 | Ref | 1.07 (0.47, 2.40) | 0.82 (0.44, 1.55) | Ref | 1.19 (0.51, 2.77) | 1.18 (0.59, 2.36) |
| % with EILV%TLC ≥ 90 | Ref | 0.64 (0.27, 1.52) | 1.46 (0.77, 2.76) | Ref | 0.60 (0.25, 1.47) | 1.57 (0.78, 3.13) |
| % with IRV ≤ 0.75 L | Ref | 1.28 (0.57, 2.87) | 2.92 (1.43, 5.97) | Ref | 1.11 (0.48, 2.55) | 2.58 (1.18, 5.63) |
| Breathlessness/V’O_2_ (Borg CR10/L/min) | Ref | 2.61 (1.95, 3.27) | 4.11 (3.59, 4.63) | Ref | 2.44 (1.80, 3.09) | 3.64 (3.10, 4.18) |
| Breathlessness/V’_E_ (Borg CR10/L/min) | Ref | 0.03 (0.02, 0.05) | 0.12 (0.11, 0.13) | Ref | 0.03 (0.02, 0.05) | 0.11 (0.10, 0.12) |
| Leg discomfort/V’O_2_ (Borg CR10/L/min) | Ref | 2.63 (1.75, 3.51) | 2.81 (2.11, 3.50) | Ref | 2.29 (1.45, 3.13) | 2.08 (1.38, 2.78) |
| Reasons for stopping CPET |  |  |  |  |  |  |
| Breathlessness | Ref | 2.04 (0.70, 5.97) | 12.74 (5.14, 31.60) | Ref | 2.04 (0.70, 5.97) | 12.23 (4.91, 30.44) |
| Leg discomfort | Ref | Ref | Ref | Ref | Ref | Ref |
| Breathlessness and leg discomfort | Ref | 1.62 (0.58, 4.51) | 4.09 (1.51, 11.09) | Ref | 1.64 (0.59, 4.57) | 3.99 (1.45, 10.96) |
| Other | Ref | 0.41 (0.11, 1.57) | 0.50 (0.10, 2.47) | Ref | 0.41 (0.11, 1.58) | 0.50 (0.10, 2.48) |

For continuous dependent variables, mixed effect linear regression models (Mixed procedure) were performed to estimate β (95% confidence interval) with random intercept accounting for study sites variability. For categorical dependent variables, mixed effect logistic regression models (Glimmix procedure) were performed to estimate odds ratios (95% confidence interval) with random intercept accounting for study sites variability. For the dependent variable "Reasons for stopping CPET", mixed effect multinomial logistic regression models (Glimmix procedure) were performed to estimate OR (95% CI) with random intercept accounting for study sites variability. Multivariable analyses were adjusted for age, sex, body mass index, and cigarette pack years.

Dependent variables reported as % predicted or LLN/ULN were adjusted for cigarette pack years and not age, sex or BMI. *Abbreviations:* see Table 2.
